# Student and expert preference intersections informing undergraduate nursing microbiology curriculum

**DOI:** 10.1093/femsle/fnag134

**Published:** 2026-10-29

**Authors:** Christian Ofume, Glenn Patriquin

**Affiliations:** School of Nursing, Faculty of Health, Dalhousie University, Halifax, Nova Scotia, B3H 4R2, Canada; Department of Microbiology and Immunology, Dalhousie University, Halifax, Nova Scotia, B3H 4R2, Canada; Department of Pathology, Dalhousie University, Halifax, Nova Scotia, B3H 4R2, Canada; Division of Microbiology, Department of Pathology and Laboratory Medicine, Nova Scotia Health, Halifax, Nova Scotia, B3H 1V8, Canada

**Keywords:** nursing, health professions, education, microbiology, curriculum

## Abstract

Education of nursing students is a serious undertaking, with impacts on the profession and patient care. Foundational microbiology curriculum may differ between programs, with no consistency in objectives. An environmental scan of publicly available data from the Canadian Association of Schools of Nursing’s list of accredited nursing schools demonstrated that approximately two thirds of programs offered a standalone microbiology course. A web-based survey of three groups (nursing students, microbiology content experts, and practicing healthcare professionals) demonstrated several highly rated microbiology topics and identified areas for which the perceived importance and preferred depth of understanding differed between groups. This small study provides insight into which topics to emphasize and de-emphasize in the nursing microbiology curriculum.

## Teaser

Triangulating the interests of content experts, healthcare professionals, and nursing students in microbiology.

## Introduction and Background

The goal of microbiology education in undergraduate health programs is to instill an understanding of microorganisms and how they impact patients, typically introducing topics such as Clinical Infectious Diseases, Antimicrobials, Infection Prevention, relevant Personal Protective Equipment (PPE), Immunology, and Vaccinology. Microbiology is typically offered as a stand-alone course in the first years of undergraduate health programs (Lee et al. 2011); however, the delivery and curriculum may differ between programs (Lukewich et al. 2023). This represents a lack of consistency in the format and delivery of microbiology education to undergraduate nursing and health-science students. This inconsistency can cause variations in content taught across Bachelor of Science in Nursing (BScN) programs, which may impact the clinical knowledge students possess when beginning practice (Abou Hashish et al. 2025).

The American Society for Microbiology’s (ASM) Microbiology in Nursing and Allied Health (MINAH) guidelines were last updated in 2018 and refer to large overarching concepts rather than specific lecture topics (Norman-McKay et al. 2018, McCall et al. 2019). Moreover, there is a paucity of literature and evidence-based data to inform the content of microbiology courses in undergraduate health programs. The competencies described by the Canadian Association of Schools of Nursing (CASN) (Canadian Association of Schools of Nursing 2022) are relevant but are overarching and may be overly broad to directly inform curriculum, as these guidelines state that baccalaureate nursing programs provide students with foundational knowledge in microbiology.

In this study, we objectively assessed publicly available syllabi from Canadian BScN programs for microbiology topics and evaluated the opinions of relevant stakeholders across Nova Scotia (nursing students, nursing instructors, registered nurses, and nurse practitioners) and across Canada (microbiology instructors, microbiologists, and infectious disease physicians) about the microbiologic concepts considered core to undergraduate nursing programs. The results of this study may aid in informing the delivery of microbiology curricula to undergraduate health programs in North America.

## Methods and design

### Environmental scan

This study’s environmental scan follows the systematic process outlined in the CADTH tool for environmental scans (https://www.cda-amc.ca/about-environmental-scanning). Using the CASN’s list of accredited nursing schools (https://www.casn.ca/accreditation), a search was conducted in May 2024 to collect publicly available data on whether microbiology was included as a stand-alone course requirement for Canadian BScN programs. Additionally, publicly available course syllabi were reviewed. To determine the availability of stand-alone microbiology courses, public-facing BScN program websites were reviewed to identify admission requirements for direct-entry and advanced standing pathways, and courses required for graduation alongside their descriptions were examined to confirm the presence of microbiology content.

Like the course review, the syllabus review included only public-facing BScN program documents. This involved analyzing learning objectives, course topics, and if applicable, laboratory topics.

### Survey development and distribution

Survey questions (supplemental material) regarding curriculum topics were derived from three sources: publicly available and personally communicated syllabi, the ASM Undergraduate Curriculum Guidelines (Boury et al. 2024), and the ASM Guidelines for Microbiology in Nursing Education (Norman-McKay et al. 2018, McCall et al. 2019). In addition to responding to preferences in curricula, participants were asked demographic questions such as highest degree attained, current professional role, clinical area of focus, practice setting, and years of experience. The survey was piloted with a convenience sample of individuals to determine flow and time commitments. The survey for participant distribution was developed using Research Electronic Data Capture (REDCap), a secure web-based application designed for research data management (https://redcap.its.dal.ca/).

A link to the web-based survey was distributed to potential participants through third-party contacts who shared the link with their respective membership or students. The invitation was sent to final-year nursing students at three universities in Nova Scotia, members of the Association for Medical Microbiology and Infectious Disease Canada, the Nurse Practitioners’ Association of Nova Scotia, and the Nurses Association of Nova Scotia. A second reminder invitation was distributed using the same method approximately 2 weeks after the initial invitation, and the survey remained open until 2 weeks after the final reminder was sent. Institutional Ethics Review Board of Dalhousie University granted approval.

### Analysis

Data analysis was performed using RStudio, Microsoft Excel, and IBM SPSS. Only complete surveys were used in the analysis. Mean Likert scale scores for topic importance (0, do not include in introductory microbiology; 5, absolutely include in introductory microbiology) and preferred depth of teaching (0, not at all in-depth; 5, very in-depth) were determined for each curriculum topic. A hierarchy was used to categorize participants with more than one profession identified, so that opinions were captured only once. Students were defined as any participant who chose “student” regardless of other selections, and content experts were defined as those who chose “instructor” or “microbiologist” regardless of their other selections. Healthcare professionals (HCPs) were defined as current registered nurses, licensed practical nurses, nurse practitioners, physicians, and any other participant who was not categorized as a student or as a content expert. If individual items were left blank, then they were omitted from the analysis.

To assess the level of agreement across the 27 curriculum topics, a one-way random effects intraclass correlation coefficient [ICC (1,1)] was used (Koo and Li 2016). Variance components were derived from a single-factor analysis of variance (ANOVA), where the curriculum topics served as the distinct groups and the mean scores from the three respondent cohorts (students, content experts, and HCPs) served as the ratings. Subgroup analysis compared the mean Likert scale scores for topic importance and depth between students, content experts, and practicing HCPs, using one way ANOVA, with a statistically significant difference defined as *P*-value of <0.05. Only those who scored all topics for either importance or depth were included in the analysis.

## Results

### Environmental scan

Using a total of 55 CASN-accredited BScN institutions, publicly available data demonstrated that 37 (67.3%) institutions offered a stand-alone microbiology course, and of those, 7 (12.7% of all courses) posted publicly available syllabi. Analysis of these syllabi revealed many similarities in the topics covered. A total of 26 topics were explored, with 18 (69.2%) of them covered by all seven programs (supplemental table).

### Survey

There was a total of 104 completed surveys used for analysis. Demographics of the respondents are shown in Table 1. Upon categorization of the participants, there was comparable representation from students (*n* = 37), content experts (*n* = 31), and HCPs (*n* = 35), with one participant abstaining from indicating their profession; therefore, their Likert data was not used. Among the participants, 94.2% attained or are attaining their first academic degree in Canada, with 67.3% of them having acquired or are acquiring their degree in Nova Scotia. Thirty-four participants were from the specialties Microbiology (*n* = 14), Infection Prevention and Control (IPAC) (*n* = 11), and Infectious Diseases (*n* = 9), with the remainder from various other disciplines. Most respondents had taken a microbiology course as a requirement for their degree (91.4%) and rate an understanding of microbiology as important to their current work (95.2%). Approximately one-third (37.5%) of respondents felt that difficult jargon caused limitations in their understanding and retaining of microbiology content.

**Table 1:** Characteristics of survey participants.

Demographic		*n* (%)*
**Degree attained or in progress**		
	Bachelor’s degree	68 (65.4)
	Master’s degree	13 (12.5)
	PhD or equivalent	8 (7.7)
	MD or equivalent	23 (22.1)
	Other degree	5 (4.8)
**Current professional role**		
	Instructor/Educator	19 (18.4)
	Physician	18 (17.5)
	Microbiologist	17 (16.5)
	Nurse practitioner	5 (4.9)
	Registered nurse	28 (27.2)
	BScN student	37 (35.9)
	Other role	11 (10.7)
**Current work setting**		
	Rural	27 (26)
	Urban	80 (76.9)
**Current work location**		
	University/Educational Institution	47 (45.2)
	Long term care/Nursing home	8 (7.7)
	In-home care	2 (1.9)
	Outpatient/Ambulatory	19 (18.3)
	Hospital inpatient	63 (60.6)
	Other	5 (4.8)
**Years of experience in profession**		
	Student (NA)	29 (27.9)
	<1	2 (1.9)
	1–5	10 (9.6)
	5–10	5 (4.8)
	10–20	24 (23.1)
	>20	33 (31.7)

*Percentages may not equal 100, as multiple responses applied to some participants.

Average scores for importance and depth of coverage (Fig. 1) revealed several highly rated topics. Overall mean importance scores of >4.5/5 were given for IPAC, Hospital-Acquired/Nosocomial Infections, Transmission of Infection, Microbial Disease, Vaccines/Immunization, Communicable Diseases, and Antimicrobials. The topics IPAC, Hospital-Acquired/Nosocomial Infections, and Transmission of infection also had an overall depth score of >4.5/5. Items with the lowest importance scores (≤3.5/5) included four laboratory topics, Bioterrorism, and History of Microbiology.

**Figure 1:**
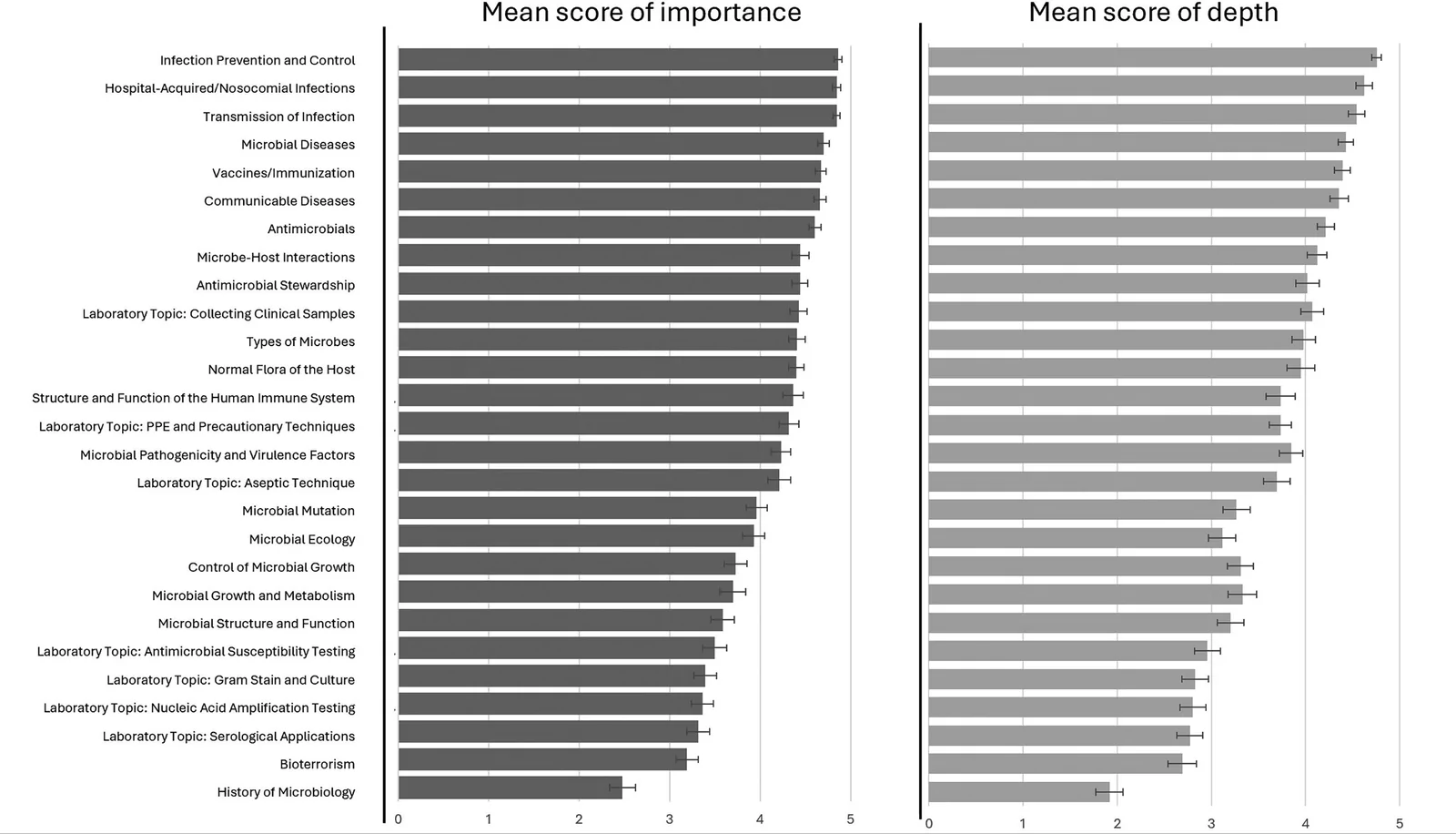
Overall mean Likert scale (0–5) scores for each microbiology topic for introductory nursing programs, based on importance (dark gray bars) and depth (light gray bars). Standard error represented by error bars.

A one-way random effects ICC (1,1) was calculated (Table 2). Topic importance scores showed good agreement, with an ICC of 0.77 (95% CI [0.62, 0.88]), while topic depth scores showed moderate agreement among the cohorts, with an ICC of 0.61 (95% CI [0.41, 0.78]). For most topics, especially those among the higher- and lower-scoring topics, differences between groups of participants were not significant. Nine topics showed significant mean score differences in importance, with experts scoring all nine topics lower than students or HCPs did (Table 3a). Significant differences between groups in topic depth were found in 17 topics, with students and HCPs consistently preferring greater depth than content experts did (Table 3b).

**Table 2:** Intraclass Correlation Coefficients demonstrating likeness between participant groups in scores of importance and depth.

	ICC value	95% CI	Agreement level*
Importance	0.77	[0.62–0.88]	Good [moderate-excellent]
Depth	0.61	[0.41–0.78]	Moderate [poor-good]

ICC, intraclass correlation coefficient; CI, confidence interval. *Based on previously-described guidelines (Koo and Li 2016).

**Table 3:** Significantly different mean scores (0–5) for (A) importance and (B) depth of microbiology topics for introductory nursing courses.

A				
Topic	Mean student score (SD) (*n* = 36)	Mean expert score (SD) (*n* = 29)	Mean HCP score (SD) (*n* = 31)	*P*-value
History of Microbiology	2.28 (1.43)	2.14 (1.25)	3.16 (1.57)	0.011
Microbial Growth and Metabolism	4.14 (1.31)	3.00 (1.41)	3.90 (1.38)	0.003
Microbial Pathogenicity and Virulence Factors	4.67 (0.59)	3.90 (1.24)	4.10 (1.19)	0.008
Microbial Mutation	4.31 (0.98)	3.59 (1.21)	3.94 (1.26)	0.046
Structure and Function of the Human Immune System	4.86 (0.35)	3.90 (1.45)	4.23 (1.28)	0.002
Microbe–Host Interactions	4.83 (0.45)	4.10 (1.11)	4.42 (1.03)	0.005
Communicable Diseases	4.83 (0.45)	4.41 (0.91)	4.74 (0.45)	0.024
Vaccines/Immunization	4.92 (0.28)	4.41 (0.83)	4.61 (0.56)	0.003
Laboratory topic: Aseptic Technique	4.58 (0.73)	3.79 (1.54)	4.29 (1.30)	0.036
**B**				
**Topic**	**Mean student score (SD) (*n* = 33)**	**Mean expert score (SD) (*n* = 27)**	**Mean HCP score (SD) (*n* = 30)**	***P*-value**
History of Microbiology	1.58 (1.20)	1.48 (1.25)	2.47 (1.46)	0.008
Microbial Ecology	2.94 (1.09)	2.56 (1.37)	3.70 (1.34)	0.003
Microbial Growth and Metabolism	3.61 (1.20)	2.26 (1.40)	3.60 (1.45)	<.001
Microbial Structure and Function	3.85 (1.09)	2.37 (1.39)	3.60 (1.28)	<.001
Microbial Pathogenicity and Virulence Factors	4.33 (1.02)	3.00 (1.41)	3.70 (1.32)	<.001
Microbial Mutation	3.55 (1.30)	2.56 (1.31)	3.50 (1.25)	0.006
Structure and Function of the Human Immune System	4.70 (0.64)	3.37 (1.42)	4.13 (1.14)	<.001
Microbe–Host Interactions	4.55 (0.75)	3.41 (1.28)	4.07 (1.11)	<.001
Control of Microbial Growth	3.33 (1.45)	2.41 (1.42)	3.87 (1.04)	<.001
Transmission of Infection	4.88 (0.33)	4.48 (.64)	4.80 (0.48)	0.007
Infection Prevention and Control	4.82 (0.53)	4.15 (1.20)	4.77 (0.77)	0.006
Hospital-Acquired/Nosocomial Infections	4.76 (0.56)	4.19 (1.08)	4.57 (0.94)	0.042
Bioterrorism	2.94 (1.56)	1.70 (1.24)	3.30 (1.15)	<.001
Communicable Diseases	4.67 (0.74)	3.74 (1.20)	4.57 (0.68)	<.001
Vaccines/Immunization	4.79 (0.55)	3.96 (0.85)	4.43 (0.73)	<.001
Laboratory topic: Aseptic Technique	4.39 (1.12)	2.96 (1.81)	4.10 (1.37)	<.001
Laboratory topic: PPE & Precautionary Techniques	4.36 (1.19)	3.19 (1.64)	4.17 (1.34)	0.004

SD, standard deviation.

## Discussion

Our study offers valuable insight into microbiology curriculum for nurses by triangulating preferred topics from nursing students, content experts, and HCPs. These insights may be helpful in creating or updating syllabi for introductory microbiology specifically for the health professions, in conjunction with previously published guidelines (Norman-McKay et al. 2018, McCall et al. 2019).

A noteworthy finding among the topics covered in Canadian microbiology courses was the agreement on included topics (i.e. those featured in all seven syllabi), which comprised a mixture of taxonomic topics, cellular-level instruction on microorganisms, and related practical healthcare topics. Only two of the seven reviewed syllabi explicitly mentioned Microbial Ecology, and Antimicrobial Stewardship. The latter topic is notable because it is recommended as a focus for better student understanding (Manning et al. 2022), though both topics are likely embedded within other lectures throughout the courses.

Scores for topic importance highlighted subject matter that could be directly applied to clinical and hospital experiences. Our results corroborate work by Durrant et al. (2017), who found that the top three topics by importance were Transmission of Infection, IPAC, and HAIs. We build on their findings, confirming the high importance of these topics among students, content experts, and HCPs. Interestingly, relatively low-scoring topics were also consistent with those ranked by the participants in the Durrant et al. study. Topics perceived as having relatively low importance in both studies included Bioterrorism, Gram Stain, and Nucleic Acid Amplification Testing, even though the latter two topics are of critical importance in the diagnostic microbiology laboratory, and an understanding of such processes is recommended in the MINAH guidelines for Laboratory Skills (Norman-McKay et al. 2018, McCall et al. 2019).

Perhaps what is most revealing about this study is the agreement between groups on the microbiology topics of highest importance. Topics such as those relating to IPAC, Transmission of Organisms, HAIs, Antimicrobials and Antimicrobial Stewardship, Infectious Diseases, and Vaccines/Immunization scored as highly important among all groups. Topics identified as important by students and HCPs included the Immune System, Microbe–Host Interactions, and Aseptic Technique, highlighting areas that could be better emphasized by instructors. For 17 topics, content experts scored the desired depth of coverage lower than students and HCPs did (whose scores were mostly aligned with each other). This may indicate that content experts underestimate what students and HCPs perceive as required knowledge; furthermore, in areas where instructors feel satisfied delivering broad, overarching lessons, students and HCPs seek a deeper understanding.

The observed ICC values demonstrate good agreement for topic importance and moderate agreement for topic depth across the curriculum. This indicates generally a shared consensus among students, content experts, and practicing HCPs regarding which clinical themes hold priority in training. The lower agreement observed for topic depth suggests that while all three groups agree on what is important to learn, there is more divergence regarding how comprehensively those topics need to be covered. These findings align with educational literature showing that there is often agreement on broad core competency domains, but differing opinions on granularity and depth based on real-world clinical demands (Frank et al. 2010; Thomas et al. 2022).

Survey responses revealed that jargon limited comprehension and learning for 37.5% of respondents during microbiology instruction. It is possible that students enrolled in microbiology may not have begun healthcare- or nursing-specific coursework at the time of enrolment, which could cause a limited understanding of medical terminology and abbreviations while concurrently learning complex microbiological processes (Nadarajah et al. 2021). More specifically, Zukswert et al. (2019) noted that students struggle most with jargon introduced during molecular structure units, concepts that are fundamental to the microbiology curriculum. To address these barriers, student comprehension may be enhanced by using inclusive language, an approach suggested by Nadarajah et al. (2021), to better align instruction with students’ current educational and healthcare experience levels.

Participants provided insightful and, at times, opposing free-text comments regarding introductory microbiology in nursing education. Several comments focused on the need to understand the practical concepts of PPE and clinical sample collection, while others specifically recommended teaching these concepts later in nursing training. Similarly, one participant acknowledged the importance of most topics presented, suggesting that many are covered in other courses or built into later training. One commenter elaborated on the desire to understand laboratory processes (i.e. what happens to a sample once submitted), which was somewhat surprising given the low importance scores for the Gram stain and nucleic acid amplification testing in the surveys. Reconciling these two opposing ideas may point to a desire to understand laboratory practices but stop short of developing the technical skills oneself. Finally, some comments suggested that the primary focus of instruction should be on topics directly related to hands-on nursing, as well as knowing how to responsibly educate patients and other healthcare workers within their scope of practice. This highlights the importance of not only teaching learners microbial concepts but also equipping them with the tools to confidently counsel patients and families on these topics.

## Limitations

Our study is not without limitations. We analyzed only publicly available syllabi, which may not represent all methods of teaching microbiology to BScN students (e.g. as components of broader biology or nursing-specific courses). Voluntary surveys may have an element of selection bias, whereby extreme opinions may be those most represented. Additionally, bias may have been introduced unpredictably through the ordering of topics listed in the survey. Further, it is difficult to draw concrete conclusions from the findings due to the response rate, though trends are consistent with the work of others, providing an element of external validation. This study provides data to expand upon with future work.

## Conclusion

Ultimately, our study demonstrates commonalities in microbiology topics that are thought to be important by three distinct groups: nursing students, practicing health professionals, and content experts. Opinions from these groups mostly align with overarching themes in prior guidelines (Norman-McKay et al. 2018) and with Canadian Nurses Association Exam Competencies and Blueprints (Canadian Nurses Association n.d.). These themes are mostly related to understanding Normal Flora, Infectious Diseases, Principals of Infection Prevention (including Immunization), and Antimicrobials (including Antimicrobial Stewardship). Future iterations of a microbiology for nursing curriculum should focus on emphasizing subjects of high importance and de-emphasizing topics thought to be of little value to students, nurses, and instructors alike.

## Supplementary Material

fnag134_Supplemental_File

## Data Availability

Data are available upon request to the authors.
