# Supplementary material for: Student and expert preference intersections informing undergraduate nursing microbiology curriculum
Source: FEMS Microbiol Lett. 2026 Oct 29;373:fnag134. doi: 10.1093/femsle/fnag134 (PMC13750152; doi:10.1093/femsle/fnag134)
Supplement: Supplementary file 1 [file fnag134_supplemental_file.docx]

**Section 1:**

**On a scale of 0-5, rate how important it is to include the following topics in the microbiology course for first-year nursing students?**

**0 being do not include and 5 being absolutely include.**

|  | 0...............................5 |
| --- | --- |
| 1. History of Microbiology |  |
| 2. Types of Microbes |  |
| 3. Microbial Ecology (e.g., the relationships between microorganisms and their environment.) |  |
| 4. Microbial Growth and Metabolism |  |
| 5. Normal Flora of the Host |  |
| 6. Microbial Structure and Function |  |
| 7. Microbial Pathogenicity and Virulence Factors |  |
| 8. Microbial Mutation |  |
| 9. Structure and Function of the Human Immune System |  |
| 10. Microbe-Host Interactions (e.g., the human body’s immune response to microorganisms.) |  |
| 11. Microbial Diseases |  |
| 12. Control of Microbial Growth (outside of the human body) |  |
| 13. Antimicrobials |  |
| 14. Antimicrobial Stewardship [e.g., how the appropriate use of antimicrobials (including antibiotics) can maximize both their current and future effectiveness” (CNA, 2023).] |  |
| 15. Transmission of Infection |  |
| 16. Infection Prevention and Control |  |
| 17. Hospital-Acquired/Nosocomial Infections |  |
| 18. Bioterrorism [e.g. “terrorism that causes death, injury, or illness by the intentional release of viruses, bacteria, fungi, or toxins.” (Lee & Kim, 2023).] |  |
| 19. Communicable Diseases (infections relevant to Public Health) |  |
| 20. Vaccines/Immunization |  |
| 21. Laboratory Topics including: |  |
| a. Aseptic Techniques |  |
| b. Collecting Clinical Samples |  |
| c. PPE and Precautionary Techniques |  |
| d. Gram Stain and Culture |  |
| e. Antimicrobial Susceptibility Testing |  |
| f. Serological Applications (AB & Ag detection) |  |
| g. Nucleic Acid Amplification Testing (PCR) |  |

Comments/Recommendations:

**Section 2:**

**Consider that the following topics are included in a first-year nursing microbiology course, on a scale of 0-5, *how in-depth* should the following topics be covered?**

**0 not in-depth at all and 5 very in-depth.**

|  | 0……………………5 |
| --- | --- |
| 1. History of Microbiology |  |
| 2. Types of Microbes |  |
| 3. Microbial Ecology (e.g., the relationships between microorganisms and their environment.) |  |
| 4. Microbial Growth and Metabolism |  |
| 5. Normal Flora of the Host |  |
| 6. Microbial Structure and Function |  |
| 7. Microbial Pathogenicity and Virulence Factors |  |
| 8. Microbial Mutation |  |
| 9. Structure and Function of the Human Immune System |  |
| 10. Microbe-Host Interactions (e.g., the human body’s immune response to microorganisms.) |  |
| 11. Microbial Diseases |  |
| 12. Control of Microbial Growth (outside of the human body) |  |
| 13. Antimicrobials |  |
| 14. Antimicrobial Stewardship [e.g., “how the appropriate use of antimicrobials (including antibiotics) can maximize both their current and future effectiveness” (CNA, 2023).] |  |
| 15. Transmission of Infection |  |
| 16. Infection Prevention and Control |  |
| 17. Hospital-Acquired/Nosocomial Infections |  |
| 18. Bioterrorism [e.g. “terrorism that causes death, injury, or illness by the intentional release of viruses, bacteria, fungi, or toxins” (Lee & Kim, 2023).] |  |
| 19. Communicable Diseases (infections relevant to Public Health) |  |
| 20. Vaccines/Immunization |  |
| 21. Laboratory Topics including: |  |
| a. Aseptic Techniques |  |
| b. Collecting Clinical Samples |  |
| c. PPE and Precautionary Techniques |  |
| d. Gram Stain and Culture |  |
| e. Antimicrobial Susceptibility Testing |  |
| f. Serological Applications (AB & Ag detection) |  |
| g. Nucleic Acid Amplification Testing (PCR) |  |

Comments/Recommendations:

**Demographic Information**

1. Education completed or in progress (check all that apply)

- Bachelor's Degree
- Master's Degree
- PhD or equivalent
- MD or equivalent
- Other: ____________

2. Current Profession or Roles (check all that apply)
   - Instructor/Educator
   - Physician
   - Microbiologist
   - Nurse Practitioner
   - Registered Nurse
   - BScN Student
   - Other: ____________
3. Years of Experience in Profession
   - Student
   - < 1
   - 1-5
   - 5-10
   - 10-20
   - > 20
4. Country where first academic degree was/will be received
   - drop down country list
5. Province where first academic degree was/will be received (if in Canada)
   - drop down province list
   - N/A option
6. Current location where your work/placement takes place (check all that apply)
   - Rural area
   - Urban area
7. Current environment where most of your work/placement takes place
   - University/Education Institution
   - Long-term care or nursing home
   - In-home
   - Outpatient/Ambulatory Clinics
   - Hospital Inpatients
   - Other (specify)
7. Specialty or area of most worked position (drop down menu)
   - Addictions
   - Antimicrobial Stewardship
   - Anesthesiology
   - Allergy/Immunology
   - Cardiology
   - Cardiovascular Surgery
   - Clinical Instructor
   - Corrections
   - Critical Care
   - Dermatology
   - Emergency
   - Endocrinology
   - Family Medicine
   - Gastroenterology
   - Genetics
   - General Medicine
   - General Surgery
   - Gerontological
   - Hematology
   - Infectious Diseases
   - Infection Prevention and Control
   - Internal Medicine
   - Interventional Radiology
   - Lecturer/Professor
   - Long-Term Care
   - Medical-Surgical
   - Mental Health
   - Microbiology
   - Minor Procedures
   - Neonatal ICU
   - Neonatal Labour and Delivery
   - Neonatal Surgery
   - Neurology
   - Nephrology
   - Neurosurgery
   - Nuclear Medicine
   - OB/GYN
   - Occupational Health and Wellness
   - Oncology
   - Ophthalmology
   - Oral-Maxillofacial Surgery
   - Orthopedic Surgery
   - Operating Room Nurse
   - Otolaryngology
   - Pathology
   - Pediatrics
   - Pediatric ICU
   - Plastic Surgery
   - Primary Care
   - Psychiatry
   - Public Health
   - Radiology
   - Research
   - Respirology
   - Rheumatology
   - School
   - Seniors Home
   - Surgical Oncology
   - Sports Medicine
   - Toxicology
   - Thoracic Surgery
   - Travel
   - Urology
   - Other (specify)

**4 Post-Survey Questions**

1. Have you taken a microbiology course as a requisite to the degree(s) applicable to your profession

(Yes or No)

2. How many years ago did you complete your introductory microbiology course?
   a. < 1
   b. 1-5
   c. 5-10
   d. 10-20
   e. > 20
3. Is an understanding of microbiology important in your current profession?

(Yes or No)

4. Did difficult terminology (e.g. jargon) cause limitations when understanding and retaining microbiology content?

(Yes, No, or N/A)

**Table. Topics covered in publicly available syllabi of Canadian BScN programs.**

| Number of syllabi with topics listed (n=7) | Topics listed in syllabi |
| --- | --- |
| 7 | Types of Microorganisms  Microbial Classification  Normal Microbiota  Microbial Structure  Microbial-Host Interactions  Infectious Diseases  Enzymes  Capsules and Escaping Phagocytosis  Identification of Microorganisms  Disease Prevention  Vaccination  Nosocomial Infections and Infection Control  MRSA, VRE, Clostridioides difficile, CPEs, TB, Respiratory Viruses  Microbial Growth and Metabolism  Control of Microbial Growth  Antimicrobials  Antimicrobial Resistance  Collecting Clinical Samples |
| 6 | Toxins, LPS, Appendages  Nosocomial Organisms vs. Community/Environment  Aseptic Technique  PPE and Precaution Techniques |
| 5 | Microbial Transmission – Chain of Infection  Koch’s Postulates |
| 2 | Microbial Ecology  Antimicrobial Stewardship |

MRSA, methicillin-resistant *Staphylococcus aureus*; VRE, vancomycin-resistant *Enterococcus*; CPEs, carbapenemase-producing *Enterobacterales*; TB, tuberculosis; LPS, lipopolysaccharide.
